# Flow Diverters for Intracranial Aneurysms

**DOI:** 10.1155/2014/415653

**Published:** 2014-05-20

**Authors:** Yazan J. Alderazi, Darshan Shastri, Tareq Kass-Hout, Charles J. Prestigiacomo, Chirag D. Gandhi

**Affiliations:** Division of Endovascular Neurosurgery, Department of Neurological Surgery, Rutgers University, New Jersey Medical School, 90 Bergen Street, Suite 8100, Newark, NJ 07101, USA

## Abstract

Flow diverters (pipeline embolization device, Silk flow diverter, and Surpass flow diverter) have been developed to treat intracranial aneurysms. These endovascular devices are placed within the parent artery rather than the aneurysm sac. They take advantage of altering hemodynamics at the aneurysm/parent vessel interface, resulting in gradual thrombosis of the aneurysm occurring over time. Subsequent inflammatory response, healing, and endothelial growth shrink the aneurysm and reconstruct the parent artery lumen while preserving perforators and side branches in most cases. Flow diverters have already allowed treatment of previously untreatable wide neck and giant aneurysms. There are risks with flow diverters including in-stent thrombosis, perianeurysmal edema, distant and delayed hemorrhages, and perforator occlusions. Comparative efficacy and safety against other therapies are being studied in ongoing trials. Antiplatelet therapy is mandatory with flow diverters, which has highlighted the need for better evidence for monitoring and tailoring antiplatelet therapy. In this paper we review the devices, their uses, associated complications, evidence base, and ongoing studies.

## 1. Introduction

During recent decades, endovascular treatment of cerebrovascular aneurysms has evolved to include unassisted coil embolization techniques, whose efficacy and safety are supported by class-1-evidence, assisted coil embolization techniques, and newly developed techniques using flow diverters [1]. While the various coil embolization techniques, including balloon assisted and stent assisted coiling, are targeted towards the aneurysm sac, flow diverters represent a paradigm shift with the intervention carried out in the parent artery [2, 3]. Flow diverter aneurysm embolization can be combined with coil embolization, further expanding the options available to clinicians and patients [3].

Flow diverters were first tested in untreatable aneurysms or those that had failed previous endovascular therapy [2]. With the approval of these devices in the USA, Europe, and other countries experience with “off-label” uses is evolving. In this paper we review the use of flow diverters for treatment of intracranial cerebral aneurysms. We review the putative mechanism of action, the technical features of devices and their uses, and the evidence for efficacy and safety of flow diverters for intracranial aneurysms.

## 2. Flow Diversion and Mechanism of Action

Flow diverters are stent-like devices that are deployed endovascularly to treat aneurysms. Conceptually, flow diverters allow endoluminal reconstruction rather than endosaccular filling. Flow diverters take advantage of changing the parent artery/aneurysm sac interface, for example, altering in-flow and out-flow jets, to induce aneurysm thrombosis. Intrasaccular thrombosis ensues after device deployment. Subsequent neointimal overgrowth covers the stent reconstructing the parent artery and eliminating the aneurysm/parent vessel interface. This process usually spares the origins of perforators [4, 5]. Furthermore, when used for fusiform aneurysms these processes allow reconstruction of a smooth endothelial covered channel in continuation with the parent artery [4]. These features are thought to allow for durable reduction in rupture rates. With time, the aneurysm shrinks and collapses around the device construct relieving symptoms from mass effect [2]. The thrombosis and associated inflammation of the aneurysm may be accompanied by temporary perianeurysmal edema in surrounding brain tissue [6]. In summary, flow diverters take advantage of hemodynamics, thrombosis, inflammation, healing, and endothelial regrowth to achieve endoluminal reconstruction and aneurysm obliteration.

As opposed to coil embolization techniques, flow diverter techniques cause aneurysms to occlude over time rather than immediately at the end of the procedure. This explains why aneurysm occlusion rates continue to increase between 6 and 12 months with flow diverters [3, 7]. Side branches, such as the ophthalmic artery with internal carotid flow diverters, may remain patent or be occluded after flow diverter implantation (Figure 3) [8]. Similarly, perforators such as those from the middle cerebral artery or those from the basilar artery usually remain patent; however, occlusions may occur [5, 9]. The incidence, clinical relevance, and risk factors for these occlusions are areas of ongoing research.

**Figure 1:**
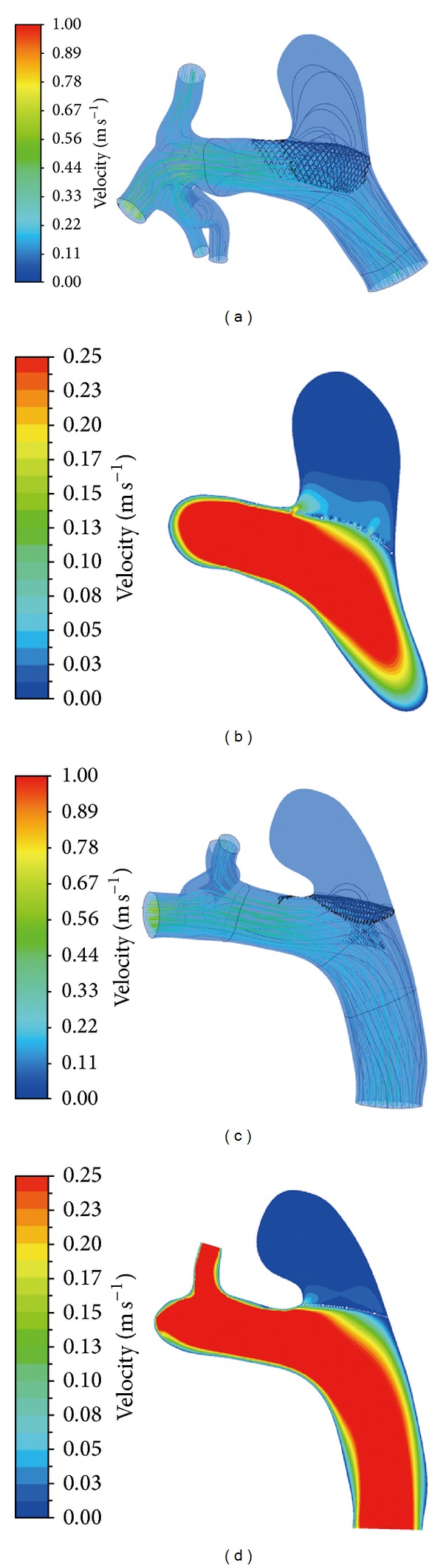
Computational fluid dynamics simulation based on micro-CT metal coverage measurement with in vivo flow diverter deployment. Inflow stream of the aneurysm sac and streamlines in <35% metal coverage ((a) and (b)) and >35% metal coverage ((c) and (d)) situations demonstrating lower mean inflow velocity with high metal coverage. Modified from [56].

The terms porosity, metal coverage, and pore density are used to describe device and deployment features that are important for flow diverter efficacy. The terms porosity and metal coverage are related. Porosity is defined as the proportion of the open metal-free area to the total stent area and metal coverage is the closed metal-covered area divided by the total stent area. Occasionally porosity or metal coverage is used to refer specifically to the area across the aneurysm neck. Some authors have termed this part of the stent the free stent segment [10]. Pore density is the number of pores per area (pores/mm^2^). Depending on the flow diverter, pore density may change or remain constant as the size of the diverter is increased. For example, in larger diameter flow diverters additional wire struts within the flow diverter wall are needed to maintain constant pore density [11]. Metal coverage across the aneurysm neck can be changed by vessel curvature and stent compaction during deployment [12, 13]. Experimental models have suggested that porosity is the most important factor in reducing intra-aneurysmal flow, with porosity of 60–76% being optimal (Figure 1) [14, 15].

## 3. Devices and Technique

**Figure 2:**
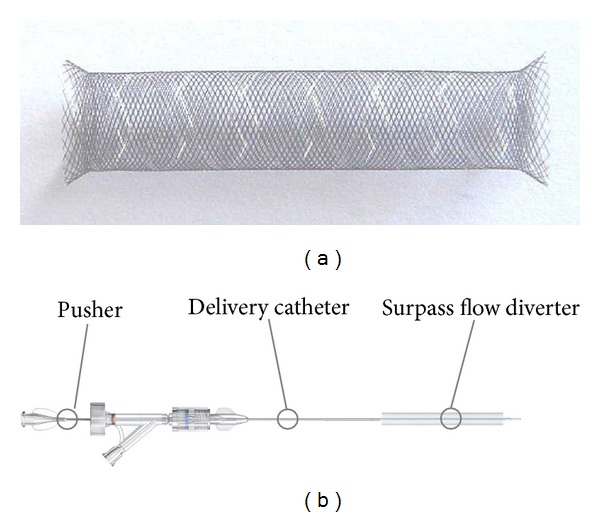
(a) The Silk flow diverter which is made of 48 braided nitinol strands with its flared ends. (b) The Surpass flow diverter which is made of cobalt-chromium alloy; also note the inner body that functions as a delivery wire. Reproduced with permission from (1) Balt Extrusion, Montmorency, France, and (2) Stryker Neurovascular.

**Figure 3:**
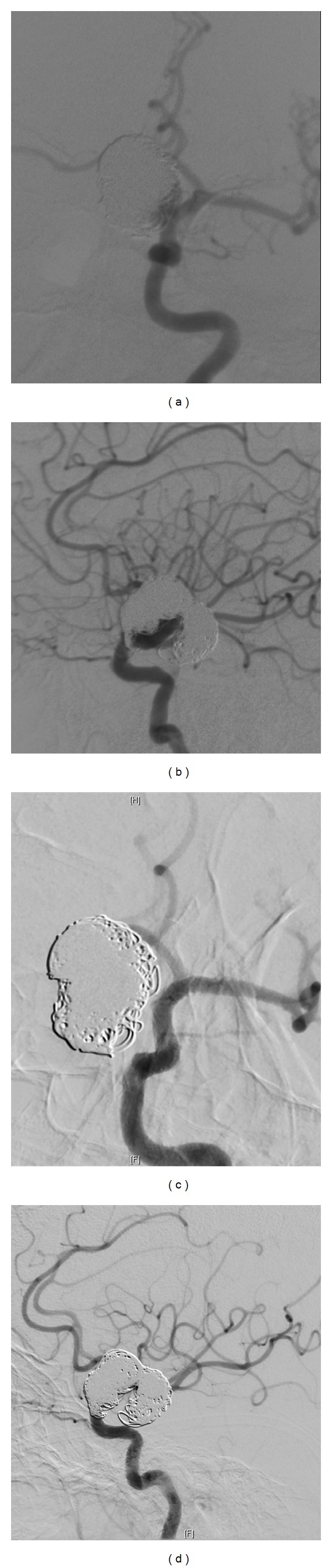
Arterial phase angiograms in (a) oblique and (b) lateral projections of a large (20 mm) left supraclinoid internal carotid artery aneurysm that had recanalized after previous coil embolization. Follow-up angiogram 8 months after placement of pipeline embolization device, (c) oblique and (d) lateral projections, demonstrating complete occlusion of the aneurysm and patency of the ophthalmic artery that was covered by the flow diverter.

Currently there are three main flow diverters: the pipeline embolization device (PED; ev3/Covidien, Irvine, California) which was approved by the Food and Drug Administration (FDA) in the USA in 2011 and is Conformité Européenne (CE) marked, certifying compliance with the European Community; the Silk flow diverter (SILK; Balt Extrusion, Montmorency, France) which was CE marked approved in Europe in 2008; and the Surpass flow diverter (SURPASS; Stryker Neurovascular, Fremont, CA) which is undergoing clinical trials in the USA and was CE marked approved in Europe in 2011 (Figure 2).

### 3.1. Pipeline Embolization Device (PED)

Pipeline embolization device is made of 25% platinum and 75% nickel-cobalt chromium alloy and consists of a stent-like device with porosity of 65–70% [3]. It is available in 2.5–5 mm diameters and 10–35 mm lengths. Multiple PEDs can be telescoped over each other to achieve different lengths although this alters porosity and pore density. The device is supplied loaded within a delivery sheath. The device is compressed and elongated to 2.5 times its nominal length. This feature requires the PED to be pushed to resume its nominal shape during deployment; the device expands radially and shortens longitudinally. The delivery wire extends 15 mm distal to the PED and sometimes requires a clockwise turn to release the PED distal end. The PED is deployed through 0.027 inch inner diameter microcatheters, Marksman Catheter (ev3/Covidien) or Renegade Hi-Flo (Boston Scientific, Fremont, CA), in a process of sequential microcatheter unsheathing and stabilization/advancement of the delivery wire. Forward pressure from the delivery wire allows the PED to expand and approximate the vessel wall. After the PED is fully deployed, the microcatheter can be carefully advanced to either capture the delivery wire or reposition distally to allow deployment of additional PEDs in a telescoping or overlapping fashion [2, 3].

The safety and efficacy of the pipeline embolization device were examined in the single arm pipeline for uncoilable or failed aneurysms study (PUFS) [2]. This study included aneurysms of the internal carotid artery (ICA) from petrous through superior hypophyseal segments. The aneurysms were >10 mm in diameter with a > 4 mm neck, and were either unamenable to or had failed coiling. The primary outcome was complete aneurysm occlusion without major parent vessel stenosis. 108 aneurysms were treated in PUFS; 20% were greater than 25 mm, 44% were petrous and cavernous ICA aneurysms, and 50.9% were paraophthalmic, superior hypophyseal, or supraclinoid ICA aneurysms. Aneurysm occlusion rate was 73.6% at 6 months. Major ipsilateral stroke or neurologic death was 5.6%. Technical device deployment success rate was 99% [3]. In Europe the single arm pipeline embolization device for the intracranial treatment of aneurysms (PITA) study was conducted. However, PITA allowed medium and small aneurysms. Aneurysms had to be wide necked >4 mm, have a dome/neck ratio <1.5, or had failed previous therapy. Adjuvant coil embolization was allowed. Aneurysms were located primarily in ICA (28 aneurysms, including 4 posterior communicating artery aneurysms); 1 middle cerebral artery and 2 vertebral artery aneurysms were also treated. Aneurysm occlusion rate was 93.3%. There were no deaths. Ischemic stroke occurred in 2 patients (6.5%). Further case series have examined the use of PED for various anterior and posterior circulation aneurysms (Figure 2) [7, 9, 16–18].

### 3.2. Silk Flow Diverter (SILK)

Silk flow diverter is available in 2–5 mm diameters and 15–40 mm lengths. It has a porosity of 45–60% [19]. The SILK delivery wire has a 9 mm distal radiopaque tip. SILK is deployed via a Vasco 21 (Balt Extrusion, Montmorency, France) microcatheter 0.0236 inch (0.6 mm) inner diameter [20]. It is deployed by careful pressure on the delivery wire and microcatheter retraction. SILK can be resheathed even when up to 90% of it has deployed.

SILK is currently unavailable for clinical use in the USA. In a meta-analysis of prospective and retrospective studies using SILK, 12-month aneurysm complete occlusion rate was 81.8%: 216 out of 264 aneurysms. Ischemic complications and parent artery occlusion each occurred in 10% of patients. Aneurysm rupture rate was 3.5%, while the cumulative mortality was 4.9 [21].

In a recent meta-analysis of cerebral aneurysm treatment with PED or SILK flow diverters, the following point estimates were noted: aneurysm complete occlusion rate of 76%, mortality of 5%, and morbidity rate of 4%. Of note, as high quality studies are limited, this meta-analysis included retrospective and prospective studies [22].

### 3.3. Surpass Flow Diverter (SURPASS)

The SURPASS is available for vessels in 2.0–5.3 mm diameters and 12–50 mm lengths. It has a porosity of 70% and pore density of 21–32 pore/mm^2^ [11]. Pore density is kept relatively constant across different diameters by varying wire struts in the device from 48 to 96. SURPASS is preloaded on a delivery microcatheter (the outer body). The device has an inner body that functions as a delivery (pusher) wire. SURPASS is advanced over a 0.014 inch microwire to the target area. The delivery wire is stabilized while microcatheter retraction unsheathes the flow diverter [4]. The manufacturer recommends one device per vessel segment without telescoping or overlapping multiple devices to maintain pore density and preserve perforator and side branch patency.

There is limited data for the use of SURPASS. In a case series with variable follow-up time, 36 of 37 patients had 1 flow diverter implanted. Successful delivery occurred in all patients. Aneurysm complete occlusion rate at 6 months was 29 of 31 aneurysms (94%) for nonbifurcation aneurysms and 5 of 10 aneurysms (50%) for bifurcation aneurysms. Neurological morbidity with eventual full recovery occurred in 4 patients (10%). Additionally, one patient (3%) developed a stroke with persistent deficit and 2 patients had dissections. There were no deaths [11]. The Surpass intracranial aneurysm embolization system pivotal trial to treat large or giant wide neck aneurysms (SCENT trial) is an ongoing single arm study to examine efficacy and safety of SURPASS (Table 1) [23].

## 4. Antiplatelet Therapy

As with other endovascular stents dual antiplatelet therapy is mandatory prior to implantation of flow diverters. Most studies used aspirin 100–325 mg and clopidogrel 75 mg daily. Patients are pretreated for several days or loaded with aspirin 325–500 mg plus clopidogrel 300–600 mg hours prior to the procedure. Therapy is continued for 6 months after the procedure in most studies [7–9, 14, 18, 24]. Aspirin is typically continued indefinitely while clopidogrel may be stopped depending on angiographic and clinical results.

In published series thromboembolic complications including in-stent thrombosis have occurred on stopping clopidogrel, even after 3 months of follow-up [11]. Patients with stenosis after device implantation seem to be at a high risk of in-stent thrombosis upon discontinuation of clopidogrel [11]. The use of platelet aggregation tests and thromboelastography (TEG) to measure medication resistance is controversial [25, 26]. Additionally, there is no data to support or refute the use of ticlopidine, cilostazol, or other antiplatelet medications in patients resistant to clopidogrel undergoing flow diverter implantation. However studies examining their use for coronary stents are available [27–30]. The need for antiplatelet therapy also complicates the use of flow diverters for ruptured aneurysms in the acute period.

## 5. Follow-Up Imaging

Catheter angiography is the gold standard test to assess residual aneurysm filling. Aneurysm occlusion may take up to 12 months with flow diverters [7]. A scheme with excellent interrater reliability has been developed for flow diverters when used for saccular or fusiform aneurysms for assessing aneurysm occlusion (5 grades from 0 to 4, with 4 being complete aneurysm occlusion) and parent artery patency (3 grades a–c; no change, narrowing, and occlusion, resp.) [31]. As opposed to follow-up of coil embolization, MRI can be used to assess aneurysm thrombosis, cerebral edema, and mass effect after flow diverter therapy [6]. Hyperintensity on FLAIR and circumferential postcontrast enhancement are thought to indicate aneurysmal inflammation as a local response to flow diverter therapy. MRI has the potential to become a clinically useful tool if future studies demonstrate that this response is associated with the development or prevention of complications [6]. Further observational studies are necessary to clarify the role and utility of MRI in follow-up of aneurysms treated with flow diverters.

## 6. Periprocedural and Delayed Complications

### 6.1. Side Branch Occlusion

Placement of flow diverters across side branches is sometimes unavoidable, such as the ophthalmic artery, anterior choroidal artery, and posterior communicating artery with ICA deployment or the posterior cerebral artery and anterior inferior cerebellar artery with basilar artery implantation. Most of the time, the side branches remain patent; for example, approximately three quarters of the time in ophthalmic artery coverage, these occlusions were asymptomatic [8, 11]. Presence of an alternative collateral pathway that can take over demand seems to be more important than the size of the side branch. For example, with SURPASS none of the 12 smaller anterior choroidal arteries remained patent while 4 of 13 (31%) posterior communicating arteries developed asymptomatic loss of antegrade flow [11]. It seems reasonable to avoid overlapping multiple devices over side branches as porosity may decrease significantly in this setting; however, good quality evidence to guide this practice is lacking.

### 6.2. Perforator Occlusion

Similar to side branch occlusion perforator occlusion may occur and is thought to be related to decreased inflow into these small vessels. This complicated 1 of 31 PED uses in PITA and accounted for 1 of the 2 strokes in the study [3]. The risk of symptomatic occlusion, 3% in meta-analysis, must be taken into consideration particularly when treating basilar artery aneurysms as the rate of perforator occlusion appears to be higher [22, 32]. Flow diverters may still be placed across perforators as neointimal endothelialization usually spares perforator origins in animal models, pore diameters are large enough compared to perforator diameters, and clinical experience shows most perforators remain open [4, 5, 33, 34]. However, this risk needs to be weighed against conservative management and other treatment options. Additionally, placing multiple overlapping flow diverters across eloquent perforators should be avoided if possible as this decreases pore size [3, 5].

### 6.3. Flow Diverter Thrombosis (In-Device/In-Stent Thrombosis)

One of the most serious complications of flow diverters is in-stent thrombosis. Adequate dual antiplatelet therapy prior to device implantation and for at least several months afterwards is mandatory [2, 3, 11, 35]. Some patients are at risk of this complication when clopidogrel is discontinued at 6 months. This seems to be particularly risky in patients with residual luminal narrowing at the device site [11]. Further observational studies are necessary to clarify the incidence and risk factors for in-stent thrombosis, as are measures to reduce the risk of antiplatelet failure.

### 6.4. Intraprocedural Vessel Perforation/Rupture

Careful monitoring of distal delivery wire position and gentle manipulation are important to avoid this complication. Perforation has been noted during balloon inflation to remodel implanted PEDs as well as during wire manipulation [3, 11]. It is recommended that angioplasty, to approximate the stent to the vessel wall or ameliorate stenosis, be carried out cautiously and that the balloon be maintained within the PED when inflated rather than trying to push the proximal device open with the balloon as arterial injury has been reported with this pushing maneuver [3, 36, 37].

Current flow diverters (PED, SILK, and SURPASS) can be safely deployed intracranially with a high degree of technical success. Key features are appropriate size selection, appropriate selection of proximal and distal landing zones, good vessel wall apposition, the avoidance of side branch and perforator coverage by the device except when necessary, careful and gentle wire and catheter manipulation, and judicious use of postimplantation remodeling techniques.

### 6.5. Perianeurysmal Edema

Extension of the inflammatory process that accompanies aneurysm thrombosis can lead to cerebral edema in adjacent tissues. This has the potential to cause worsening of compressive symptoms or headache, which is transient. In a prospective MRI study, perianeurysmal edema was associated with giant aneurysms and close proximity to brain without intervening cerebrospinal fluid space. Interestingly increase in aneurysm size after treatment was not observed in these cases [6]. Of note, perianeurysmal edema has been reported with aneurysm coiling and after therapeutic parent vessel occlusion [38, 39]. The optimal preventative and therapeutic measures for this complication have not been determined. Steroids have a variable response [6].

### 6.6. Distant Infarction

Rarely have both clinically silent and symptomatic distant infarctions been observed after flow diverter implantation [2, 40]. This is thought to be due to the excessive manipulation that is sometimes necessary to deploy these devices. There is hope that as the devices become easier to deploy this complication will occur less frequently.

### 6.7. Delayed Hemorrhage

There are two types of delayed hemorrhage: intraparenchymal distant hemorrhage and subarachnoid hemorrhage, each occurring in 3% of cases [22]. Our understanding of these complications is still evolving. Distant hemorrhages ipsilateral to the flow diverter deployment are thought to be related to hemorrhagic transformation of infarcts that have occurred during the procedure [41, 42]. Delayed subarachnoid hemorrhage may occur due to degradation of aneurysm wall by enzymes triggered during aneurysm thrombosis, while acute subarachnoid hemorrhage may occur with wire perforations [11, 43, 44].

**Figure 4:**
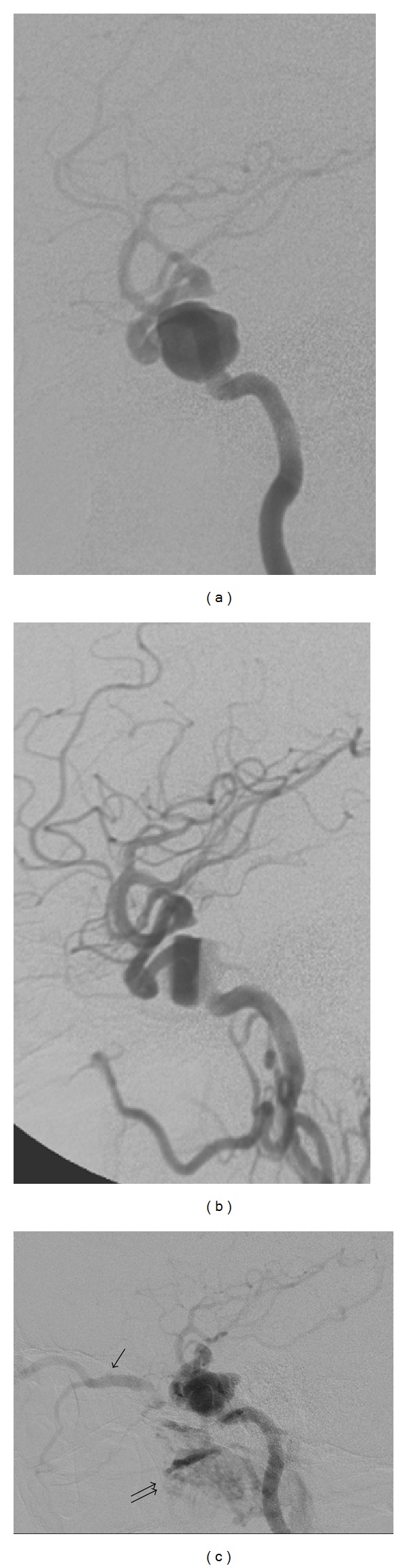
Arterial phase lateral view angiograms of a right cavernous internal carotid artery aneurysm, (a) initial pretreatment angiogram, (b) immediate contrast stasis within the aneurysm at the end of deployment of two telescoping pipeline embolization devices, and (c) carotid cavernous fistula on angiogram at 4 months after treatment done for symptoms of right eye pain, swelling, and vision loss. Note filling of aneurysm sac in (c) as well as venous drainage in the enlarged superior ophthalmic vein (arrow) and the pterygoid venous plexus (double arrows).

Another infrequent and delayed complication is carotid cavernous fistula development. This was noted in PUFS and has been noted with stent assisted coiling in the past (Figure 4) [45].

## 7. Atypical Uses: “Off-Label Uses”

Prospective observational studies have laid the foundation for clearance and approval of flow diverters for clinical use in the USA and Europe (Table 2). As with other new medical technologies, experience with off-label uses continues to develop. Posterior circulation aneurysms, which were untreatable by surgical or other endovascular means, underwent PED treatment in an Australian registry [9]. There was a 96% aneurysm occlusion rate at 12 months, 9.4% neurological complication rate that was due to perforator infarctions in all cases with complications, and no mortality in 21 patients. Perforator infarctions seem to be more common in flow diversion of basilar artery aneurysms [22].

Currently there is not enough evidence to support the use of flow diverters for bifurcation aneurysms and blister-like aneurysms although preliminary animal and clinical data on these uses has been reported with mixed results [11, 17, 46, 47].

## 8. Ongoing Clinical Trials

Initial experience with flow diverters was in aneurysms without other treatment options or aneurysms that had failed prior therapy. Several randomized controlled trials are ongoing to evaluate flow diverters for other indications (Table 1) [48–52]. Flow diverters have the potential to address high recanalization rates seen with some types of aneurysms after coil embolization [53, 54]. As experience with flow diverters increases, new iterations of devices develop, and antiplatelet regimens are refined, we may expect flow diverters to have a complication rate low enough to compete with coil embolization and surgery in aneurysms amenable to these therapies [48–52]. Finally flow diverters have been allowed in the endovascular arm of the international subarachnoid aneurysm trial II (ISAT II) [55]. This study and observational studies might provide some evidence for flow diverter use in the setting of ruptured aneurysms.

## 9. Conclusions

Flow diverters have expanded the therapeutic options for treatment of cerebral aneurysms and represent a welcomed paradigm shift. Previously untreatable intracranial aneurysms can now be safely treated. Comparative studies on efficacy and safety are underway to address the gaps in evidence for other indications. The role of flow diverters is evolving and expanding. Treatment of blister-like aneurysms, bifurcation aneurysms, small aneurysms, and aneurysmal dysplastic arterial segments with multiple small aneurysms using flow diverters requires further study to evaluate whether the benefit exceeds the risks. Lastly flow diverters use has reignited the need for research of safer and more efficacious use of antiplatelets in elective and emergent endovascular techniques.

## Figures and Tables

**Table 1:** Ongoing clinical trials involving flow diverters for intracranial aneurysms.

Trial name	Patient population	Intervention	Comparison	Outcome
Flow diversion in intracranial aneurysm treatment (FIAT) trial	Any patient with a “difficult” intracranial aneurysm in whom flow diversion is considered an appropriate if not the best yet unproved therapeutic option by the participating clinician	Flow diversion	Standard treatment of any of the following: (1) conservative management, (2) coil embolization with or without high porosity stent, (3) parent vessel occlusion, or (4) surgical clipping	Rate of successful therapy at 12 months. Success defined as complete or near complete occlusion of the aneurysm combined with a modified Rankin score less than or equal to 2

LARGE aneurysm randomized trial: flow diversion versus traditional endovascular coiling therapy (LARGE)	Patients aged 21–75 internal carotid artery aneurysms (petrous, cavernous, and paraophthalmic) with neck and fundus morphologies amenable to either traditional endovascular treatments using coils or reconstruction with the flow diversion. Aneurysm neck = or >4 mm. Fundus = or >10 mm	Flow diversion	Endovascular coil embolization	Noninferiority with regard to efficacy and safety at 180 days after procedure. Efficacy: greater than 90% aneurysm occlusion rate and stable (or decreased) aneurysm size on cross-sectional CT or MRI. Safety: absence of major neurological event or death

Endovascular treatment of intracranial aneurysm with pipeline versus coils with or without stents (EVIDENCE) trial	Unruptured saccular intracranial aneurysms larger than 7 mm	Pipeline embolization device	Endovascular coil embolization with or without balloon remodeling, with or without stent assistance	Angiographic aneurysm complete occlusion rates at 12 months

Complete occlusion of coilable intracranial aneurysms (COCOA) trial	“Coilable” aneurysms of the petrous, cavernous, and supraclinoid segments of the internal carotid artery	Pipeline embolization device	Endovascular coil embolization	Complete angiographic occlusion of the target aneurysm 180 days after treatment

Multicentre randomised trial on selective endovascular aneurysm occlusion with coils versus parent vessel reconstruction using the SILK flow diverter (MARCO POLO)	Patients with at least one documented untreated, unruptured intracranial aneurysm suitable for occlusion with an intracranial device	SILK flow diverter without coils	Endovascular coil embolization with or without balloon remodeling or stent assistance	Angiographic aneurysm complete occlusion rates at 12 months

The Surpass intracranial aneurysm embolization system pivotal trial to treat large or giant wide neck aneurysms (SCENT trial)	19–80-year-old patients with single targeted wide neck, large, or giant intracranial aneurysms of the internal carotid artery up to the terminus	Surpass flow diverter	None	Complete aneurysm occlusion without clinically significant stenosis (>50%) of parent artery at 12 months. Absence of neurological death or ipsilateral stroke at 12 months

International subarachnoid aneurysm trial II (ISAT II)	Ruptured intracranial aneurysms not included in the original ISAT study: at least one documented, intradural, and intracranial aneurysm ruptured within last 30 days. Subarachnoid hemorrhage, world federation of neurological surgery grade 4 or less. The patient and aneurysm are considered appropriate for either surgical or endovascular treatment by the treating team	Endovascular therapy with use of coils, balloon remodeling, stents, or flow diverters as per physician performing treatment	Surgical management, surgical clipping with or without bypass, and other surgical flow redirecting methods as per physician performing treatment	Poor clinical outcomes; modified Rankin scale >2 at 12 months

**Table 2:** On-label indications for flow diverters.

Flow diverter	Indication in USA	Indication in Europe
Pipeline embolization device (PED; ev3/Covidien, Irvine, California)	Patients aged 22 and older with large or giant wide-necked intracranial aneurysms in the internal carotid artery from the petrous to superior hypophyseal segments	The endovascular embolization of cerebral aneurysms

Silk flow diverter (SILK; Balt Extrusion, Montmorency, France)	Not yet FDA approved	The treatment of intracranial aneurysms in association with embolization coils

Surpass flow diverter (SURPASS; Stryker Neurovascular, Fremont, CA)	Not yet FDA approved	Saccular or fusiform intracranial aneurysms arising from a parent vessel with a diameter of ≥2 mm and ≤5.3 mm

## Abbreviations

COCOA: Complete occlusion of coilable intracranial aneurysms trial
CT: Computed tomography
EVIDENCE: Endovascular treatment of intracranial aneurysm with pipeline versus coils with or without stents trial
FIAT: Flow diversion in intracranial aneurysm treatment trial
FLAIR: Fluid attenuated inversion recovery
ICA: Internal carotid artery
ISAT II: International subarachnoid aneurysm trial II
LARGE: Large aneurysm randomized trial: flow diversion versus traditional endovascular coiling therapy
MARCO POLO: Multicentre randomised trial on selective endovascular aneurysm occlusion with coils versus parent vessel reconstruction using the Silk flow diverter
Mm: Millimeters
MRI: Magnetic resonance imaging
PED: Pipeline embolization device
PITA: Pipeline embolization device for the intracranial treatment of aneurysms
PUFS: Pipeline for uncoilable or failed aneurysms study
SCENT: The Surpass intracranial aneurysm embolization system pivotal trial to treat large or giant wide neck aneurysms
SILK: Silk flow diverter
SURPASS: Surpass flow diverter
TEG: Thromboelastography
USA: United States of America.
